# The Last Contribution of a Great Authority

**Published:** 1919-11-01

**Authors:** 


### LUNACY LAW REFORM.

### The Last Contribution of a Great Authority.

THE MEDICO-PSYCHOLOGICAL ASSOCIATION issued a Report on Lunacy Legislation, which we opened with the confident expectations of finding proposals to change the names of things, and to give new names to old things, this being in the estimation of the members of the Medico-Psychological Association, what is meant by Reform, Progress, Discovery, Research, and so forth. We were not disappointed. The Association with the unwieldy title proposes that the words "Lunacy" and "Lunatic" shall be discontinued, and the words "Mental Disease" and "Person of Unsound Mind" substituted for them. This is only a part of the important reform in the Lunacy Laws that is recommended by the Association. It would change the name of "Asylum" into "Mental Hospital," and "Pauper" into "Rate-aided," and it would have "Lunacy Legislation" called "Mental Treatment Legislation."

These able and astute gentlemen have already effected far-reaching reforms of the same character. They have dubbed themselves Psychiatrists in place of the old and descriptive titles of mad-doctors or alienists, and they are still pursuing the same fruitful line of research, and making similar discoveries. It is a noble ambition, and leads to momentous results; and its great advantage is that it is open to all. It needs no training, no experience, no costly laboratories, no days and nights of labour. All that is needed is a dictionary, and even this is usually dispensed with, for some of the greatest of these discoveries do not harmonize with their dictionary value, and it is clear that the discoverers do not know the meanings of the words they discover. The game—we mean the line of research—is so fascinating that we feel inclined to take part in it, and though we cannot pretend to the skill that the members of the Medico-Psychological Association have attained by assiduous practice, we will do our little best, and suggest that the term "Lunacy Law" should be replaced by "Medico-Psychological Law"; and the term "Asylum" by the term "Medico-Psychological Hospital." It might not do to substitute for the terms, "Lunatic, idiot, or person of unsound mind," the term "Medico-Psychologist," but it is clear that the substitution has advantages and is certainly more accurate than some of those recommended by the Medico-Psychological Association.

The twenty-four recommendations of the Association were not all of this character. A considerable part of the Report is occupied with the recommendation of "Clinics," by which it appears that the Medico-Psychological Association means more Medico-Psychological buildings, which are to be "established all over the country." We remember the same aspiration thirty years ago. It was then a fixed and settled doctrine that the proper treatment of madness, or, let us give it its proper title, of Medico-Psychological disease, is by bricks and mortar; and that if we only build plenty of asylums, we mean Medico-Psychological Institutions, "all over the country," and build them large enough, and palatial enough, we should give to madness all the treatment it is capable of receiving. Since then we have been steadily building these institutions all over the country, but the result has scarcely answered our expectations. Indeed, it may be said that, except as a palliative, the treatment of Medico-Psychological disease by bricks and mortar has failed. The remedy, in the eyes of the Medico-Psychologist is—more bricks and mortar.—C.A.M.

